# Fe65 Is Phosphorylated on Ser^289^ after UV-Induced DNA Damage

**DOI:** 10.1371/journal.pone.0155056

**Published:** 2016-05-13

**Authors:** Hannah Langlands, Peter G. Blain, Paul A. Jowsey

**Affiliations:** NIHR Health Protection Research Unit for Chemical & Radiation Threats & Hazards, Wolfson Building, Newcastle University, Newcastle upon Tyne, NE2 4AA, United Kingdom; Torrey Pines Institute for Molecular Studies, UNITED STATES

## Abstract

Fe65 undergoes a phosphatase-sensitive gel mobility shift after DNA damage, consistent with protein phosphorylation. A recent study identified Ser^228^ as a specific site of phosphorylation, targeted by the ATM and ATR protein kinases, with phosphorylation inhibiting the Fe65-dependent transcriptional activity of the amyloid precursor protein (APP). The direct binding of Fe65 to APP not only regulates target gene expression, but also contributes to secretase-mediated processing of APP, producing cytoactive proteolytic fragments including the APP intracellular domain (AICD) and cytotoxic amyloid β (Aβ) peptides. Given that the accumulation of Aβ peptides in neural plaques is a pathological feature of Alzheimer’s disease (AD), it is essential to understand the mechanisms controlling Aβ production. This will aid in the development of potential therapeutic agents that act to limit the deleterious production of Aβ peptides. The Fe65-APP complex has transcriptional activity and the complex is regulated by multiple post-translational modifications and other protein binding partners. In the present study, we have identified Ser^289^ as a novel site of UV-induced phosphorylation. Interestingly, this phosphorylation was mediated by ATM, rather than ATR, and occurred independently of APP. Neither phosphorylation nor mutation of Ser^289^ affected the Fe65-APP interaction, though this was markedly decreased after UV treatment, with a concomitant decrease in the protein levels of APP in cells. Using mutagenesis, we demonstrated that Fe65 Ser^289^ phosphorylation did not affect the transcriptional activity of the Fe65-APP complex, in contrast to the previously described Ser^228^ site.

## Introduction

Fe65 is an adaptor protein consisting of an N-terminal WW domain and two C-terminal phosphotyrosine binding domains (PTB1 and PTB2) [1]. By direct binding, Fe65 regulates the secretase-mediated processing of APP [2] and formation of multiple proteolytic APP fragments, including a large extracellular fragment, the 40–42 amino acid Aβ peptide (from the APP transmembrane region) and the APP intracellular domain (AICD). Aβ has cytotoxic potential and is likely to play an important role in the neurodegenerative processes associated with AD [3]. AICD is thought to translocate to the nucleus, in a process involving direct binding to the PTB2 domain of Fe65 [4,5], before this complex (with other co-activator proteins) activates the expression of genes including APP itself, BACE1, GSK3β, acetylcholinesterase, KAI1, WASF, BEST1 and TIP60[6–11]. Cao and Sudhof demonstrated the transcriptional activity of AICD (and full length APP) by generating fusion proteins with the GAL4 DNA binding domain. Only in the presence of overexpressed Fe65 did AICD-GAL4 or APP-GAL4 fusion proteins activate GAL4-dependent luciferase production [12]. Given the cellular consequences of aberrant APP processing and the important transcriptional role of APP/AICD, it is essential to understand mechanisms regulating these processes, for example the identification and characterisation of Fe65 post translational modifications.

Phosphorylation of both proteins contributes to the regulation of the Fe65-APP interaction. For example, direct binding between the PTB2 domain of Fe65 and the YENPTY motif of APP/AICD is impaired by phosphorylation of APP Thr^668^ [7,13]. Genetic manipulation of the Fe65-APP interaction (e.g. by mutation of Thr^668^ or by overexpression/down-regulation of Fe65) affects APP processing, with subsequent alterations in the levels of Aβ [13–16]. Mutation of a conserved cysteine in the Fe65 PTB2 domain blocks the interaction of Fe65 with APP and concomitantly abolishes the transcriptional activity of APP [[17,18] and data in the present study]. In addition, transcription induced by the Fe65-APP complex is enhanced by interaction with the c-Abl protein kinase and subsequent phosphorylation on Fe65 Tyr^547^ (within PTB2) [19].

As well as roles in the regulation of APP, Fe65 has additional roles in the cellular response to DNA damage [20–22]. Fe65 knockout mice showed elevated levels of DNA damage after exposure to etoposide or ionising radiation [20]. Mechanistically, these effects were caused (at least in part) by inefficient recruitment of the histone acetyltransferase TIP60 to sites of DNA double strand breaks (DSBs), inefficient histone H4 acetylation and defective chromatin relaxation (an essential part of DNA repair processes) [22]. Fe65 was also shown to undergo a rapid phosphatase-sensitive gel mobility shift after DNA damage, consistent with protein phosphorylation, though no specific sites were identified nor functionally characterised [20]. Protein phosphorylation after DNA damage is orchestrated by the ATM (ataxia telangiectasia mutated) and ATR (ataxia telangiectasia and Rad3-related) kinases. These kinases generally respond to different forms of DNA damage, for example ATM is activated in the presence of DNA DSBs (e.g. IR or etoposide), whereas ATR is activated by stretches of single-stranded DNA, generated during the processing of various DNA adducts and cross-links [23,24]. Active ATM/ATR phosphorylate multiple target proteins, with target sequences corresponding to serine or threonine residues followed by a glutamine, often referred to as the ‘SQ/TQ’ motif [25,26]. We have recently identified Fe65 Ser^228^ as a novel DNA damage-induced phosphorylation site and showed that blocking Ser^228^ phosphorylation (by introducing an alanine mutation) enhanced Fe65-APP transcriptional activity [17]. This suggests that DNA damage-induced phosphorylation could contribute to the complex regulatory mechanisms involved in Fe65-APP function and thus be relevant to AD.

This study aimed to further characterise the role of DNA damage-induced Fe65 phosphorylation in regulating the Fe65-APP interaction or Fe65-APP-mediated gene transcription. This study identified Fe65 Ser^289^ as a novel site of UV-induced phosphorylation, targeted by the ATM protein kinase. Neither phosphorylation nor mutation of Ser^289^ affected the Fe65-APP interaction. In addition, mutation of Fe65 Ser^289^ to alanine (thus blocking phosphorylation) did not affect the transcriptional activity of the Fe65-APP complex, in contrast to the previously described Ser^228^ site.

## Materials and Methods

### Cell lines, chemicals and treatments

HEK293 cells were obtained from PHE Culture Collections and maintained as exponentially growing cultures in Dulbecco's Modified Eagle Medium (DMEM) supplemented with 10% fetal bovine serum (FBS) and 2mM L-glutamine. The ATR inhibitor AZ20 and ATM inhibitor KU55933 (Tocris) were prepared at 10mM in DMSO. Inhibitors were added to cells at at final concentrations of 5μM (AZ20) and 10μM (KU55933) 45min prior to UV treatment, with DMSO levels maintained at 0.1% (v/v). For UV treatment, the growth medium was removed from cells, before plates were placed in a UV crosslinker (lids were removed from the culture plates) and exposed to 4-40Jm^2^ UV, as indicated in individual figures.

### Plasmids

For overexpression studies, human Fe65 with a C-terminal FLAG-Myc tag was obtained from Origene (RC202003) and Fe65 sub-cloned into expression vectors with N-terminal GFP or FLAG tags (PS100048 and PS100014, respectively, Origene). Specific mutants of Fe65 were generated using the Quikchange II XL site directed mutagenesis kit (Agilent). Primer details can be provided on request. For luciferase reporter gene assays, pMST-APP [provided by Prof Kwok-Fai Lau (The Chinese University of Hong Kong), with the kind permission of Prof Thomas Südhof (Stanford University School of Medicine, USA)], pFR-Luc (Agilent) and pRL-TK (Promega) were used.

### Immunoprecipitation, Western blotting and Antibodies

After the indicated treatments, cells were washed in cold PBS before lysis in NETN buffer (50mM Tris pH 7.6, 150mM NaCl, 1mM EDTA and 0.5% NP-40). After incubation at 4C for 15 min, lysates were cleared by centrifugation and supernatants isolated. For immunoprecipitation, cell lysates were incubated with either GFP-Trap (Chromotek, for the purification of GFP-tagged proteins) or FLAG-M2 agarose beads (Sigma, for the purification of FLAG/FLAG-tagged proteins) for 2 hours at 4C before washing three times in TBST (50mM Tris pH 7.6, 150mM NaCl and 0.2% Tween-20). For immunoprecipitation of endogenous Fe65, cell lysates were incubated overnight with 4μl of Fe65 antibody (ab91650, Abcam). For lambda phosphatase treatment, beads were resuspended in 50μl of reaction buffer and 200U of lambda protein phosphatase (P0753S, New England Biolabs) for 15 min at 30C before washing again in TBST. Beads were resuspended in an equal volume of 2X LDS sample buffer (Life Technologies) containing 2.5% 2-mercaptoethanol before heating at 95C for 5 min. Samples were analysed by Western blotting using 4–12% bis-tris gels (Life Technologies) before transferring to nitrocellulose using an iBlot machine (Life Technologies). Membranes were blocked in 2.5% BSA/TBST for 1 hour prior to overnight incubation (4C) in blocking buffer containing the following primary antibodies: GFP (2956), APP (2452), phospho-S/T-Q (‘pATM/ATR motif’, 2851), CHK1 phospho-Ser317 (12302), GAPDH (2118), all from Cell Signalling Technology and FLAG (F1804, Sigma). The primary antibody referred to as p-Fe65x in this study was raised against a phosphopeptide encompassing the previously described Ser^228^ site. Characterisation of this antibody revealed that mutation of Fe65 Ser^228^ reduced, but did not abolish, the DNA damage-induced Western blot signal after p-Fe65x detection. We hypothesised that p-Fe65x was thus recognising additional Fe65 phosphorylation sites and used this antibody and mutagenesis in the present study to identify a new Fe65 phosphorylation site. After incubation with the relevant HRP-conjugated secondary antibody, western blots were visualised using ECL Prime (GE Healthcare) and images captured using a Syngene G:Box gel documentation system. When images were quantified, ImageJ software was used, with levels of p-Fe65x normalised to Fe65 levels in the corresponding immunoprecipitate. For statistical analysis, a paired t-test (two-tailed) was used, with level of significance defined as: p < 0.05 (*), p < 0.01 (**) and p < 0.001 (***). All Western blot images are representative of at least three independent experiments.

### GFP microscopy

For analysis of GFP-Fe65 and GFP-Fe65 S289A localisation, HEK293 cells were transfected with the relevant plasmid using calcium phosphate. After 24h, cells were fixed on glass coverslips with 4% formaldehyde and stained with DAPI. Fluorescence images were captured using a Zeiss Axioscope fluorescence microscope. Data are representative of at least three independent experiments.

### Luciferase Reporter Gene Assay

The APP-dependent luciferase assay has been described previously and was performed according to our earlier study [12,17].

## Results

### Fe65 is phosphorylated on Ser^289^ after UV-induced DNA damage

**Fig 1:**
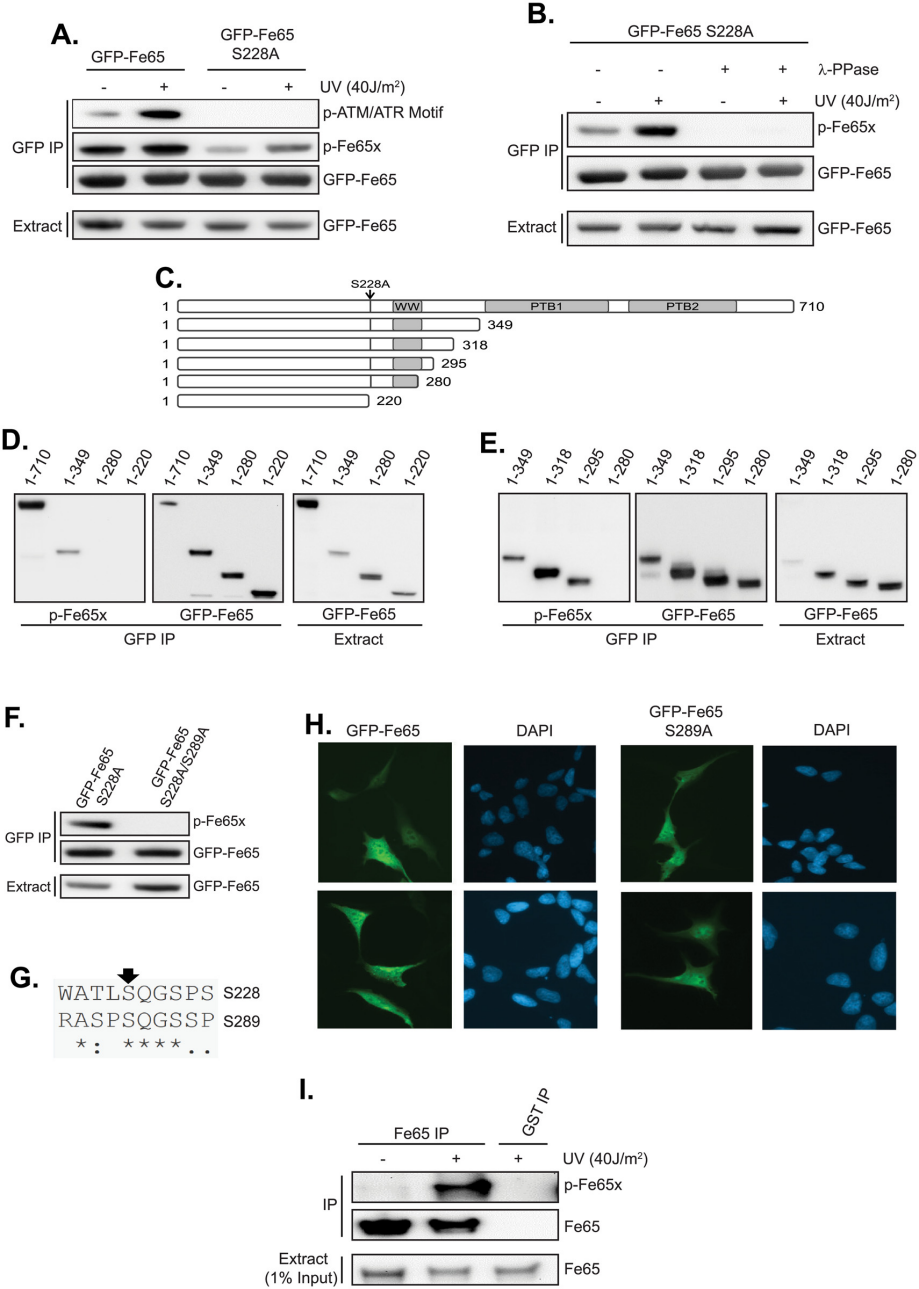
Fe65 is phosphorylated on Ser^289^ after UV-induced DNA damage. (A) HEK293 cells were transfected with GFP-Fe65 or GFP-Fe65 S228A for 24h before treatment with 40J/m^2^ UV for 2h. Cell extracts were subjected to GFP immunoprecipitation and samples analysed by Western blotting using the indicated antibodies. (B) HEK293 cells were transfected with GFP-Fe65 S228A for 24h before treatment with 40J/m^2^ UV for 2h. After GFP immunoprecipitation, each sample was split and half treated with lambda phosphatase for 15min at 30C, before western blot analysis with the indicated antibodies. (C) Schematic showing the panel of GFP-Fe65 S228A deletion mutants, which were transfected into cells in (D) and (E) before UV-treatment (40J/m^2^ 2h), GFP immunoprecipitation and Western blotting with the indicated antibodies. (F) GFP-Fe65 S228A or GFP-Fe65 S228A/S289A were transfected into HEK293 cells for 24h before treatment with 40J/m^2^ UV (2h). After GFP immunoprecipitation, samples were analysed by Western blotting with the indicated antibodies. (G) Alignment of the amino acid sequence surrounding Ser^228^ and Ser^289^. (H) Fluorescence microscopy showing cellular localisation of GFP-Fe65 and GFP-Fe65 S289A. Images are representative of at least three independent experiments. (I) Endogenous Fe65 was immunoprecipitated from untreated or UV-treated cells and samples analysed by Western blotting with the indicated antibodies. GST antibody was used in negative control immunoprecipitations from UV-treated cells.

As part of an earlier study, we identified Fe65 Ser^228^ as a DNA damage-induced site of phosphorylation using an anti-ATM/ATR motif antibody [raised against a library of peptides phosphorylated on Ser/Thr followed by Gln (SQ/TQ)–the ATM/ATR ‘consensus sequence’]. We attempted to generate a phosphospecific antibody to Ser^228^ and tested the reactivity/specificity of this antibody in immunoprecipitations. Throughout the manuscript, this antibody is referred to as p-Fe65x. Cells were transfected with GFP-Fe65 or GFP-Fe65 S228A and Fe65 immunoprecipitated from untreated cells or UV-treated cells (to induce DNA damage). As shown in Fig 1A and (1A in S1 Fig), immunoprecipitated GFP-Fe65 was detected by the pATM/ATR motif antibody and the signal was markedly increased after DNA damage. This signal was completely abolished by mutation of Ser^228^, consistent with our previously published data. The same samples were analysed using the p-Fe65x antibody. Again, GFP-Fe65 was detected by this antibody and the signal was increased after DNA damage. However, when Ser^228^ was mutated, the p-Fe65x signal was reduced but not completely abolished, and was still increased after UV. This strongly suggests that p-Fe65x recognises Ser^228^, as well as additional DNA damage-induced phosphorylation site(s). To confirm that p-Fe65x was binding other phosphorylation sites in GFP-Fe65 S228A, immunoprecipitates were analysed in the presence and absence of λ-phosphatase. As shown in Fig 1B, p-Fe65x detected GFP-Fe65 S228A and the signal was increased after UV. Importantly, this signal was completely abolished when GFP-Fe65 S228A immunoprecipitates were treated with λ-phosphatase. These data confirm that p-Fe65x is recognising one or more additional UV-induced phosphorylation sites in GFP-Fe65 S228A.

Given that the p-Fe65x antibody was raised against a peptide encompassing a phospho-SQ motif, and the signal was increased after DNA damage, we hypothesised that another phospho-SQ site was being recognised by this antibody. To help identify the specific SQ site (or sites) being recognised by p-Fe65x, a series of Fe65 deletion mutants (Fig 1C) were immunoprecipitated from UV-treated cells and tested for p-Fe65x reactivity. Importantly, each deletion mutant also had Ser^228^ mutated to Ala, to prevent p-Fe65x recognising this site. As shown in Fig 1D, Fe65 1–349 was detected by p-Fe65x but Fe65 1–280 and 1–220 were not (compare lane 2 with lanes 3/4 in left hand panel). To confirm phosphorylation of Fe65 S228A 1–349, we demonstrated that p-Fe65x signal was completely abolished by phosphatase treatment of UV-treated GFP-Fe65 S228A 1–349 immunoprecipitates (1B in S1 Fig). This means that the phosphorylation site being detected by p-Fe65x is within the region 280–349. Next, a further series of deletion mutants targeting this region were constructed, and immunoprecipitated from UV-treated cells. As shown in Fig 1E, 1–295 was detected by p-Fe65x, whereas 1–280 was not. Analysis of the Fe65 protein sequence showed that there was only one SQ site in this region—Ser^289^. Therefore, Ser^289^ was mutated in GFP-Fe65 S228A to generate the S228A/S289A double phosphomutant. These Fe65 variants were immunoprecipitated from UV-treated cells and tested for reactivity with p-Fe65x. As shown in Fig 1F, p-Fe65x recognised GFP-Fe65 S228A but this signal was totally lost when Ser^289^ was additionally mutated. This confirms that Ser^289^ is a novel site of phosphorylation (likely to be targeted by ATM/ATR due to SQ sequence) and that the p-Fe65x antibody is able to detect both Ser^228^ and Ser^289^. Analysis of the sequence context of these two phosphorylation sites reveals significant similarity, with an identical SQGS sequence present in both (Fig 1G). This is likely to explain why p-Fe65x recognises both Ser^228^ and Ser^289^. To validate the mutagenesis data, it was important to show that the loss of p-Fe65x reactivity in the S228A/S289A mutant was not simply due to a mutation-induced change in cellular localisation. Previous studies have showed that Fe65 is present in both the cytoplasm and nucleus (with ATM and ATR being predominantly nuclear). Using fluorescence microscopy, it was demonstrated that mutation of Ser^289^ in GFP-Fe65 did not affect cellular localisation, with Fe65 and S289A being present in both the cytoplasm and nucleus (Fig 1H). The localisation of Fe65 is consistent with previously published data [27–29]. Together, these data confirm that Fe65 Ser^289^ is a novel DNA damage-inducible phosphorylation site. Importantly, it was demonstrated that p-Fe65x reacted with immunoprecipitated endogenous Fe65, with the western blot signal markedly increased after UV (Fig 1I). This shows that endogenous Fe65 is phosphorylated after UV, likely on both Ser^228^ and Ser^289^.

**Fig 2:**
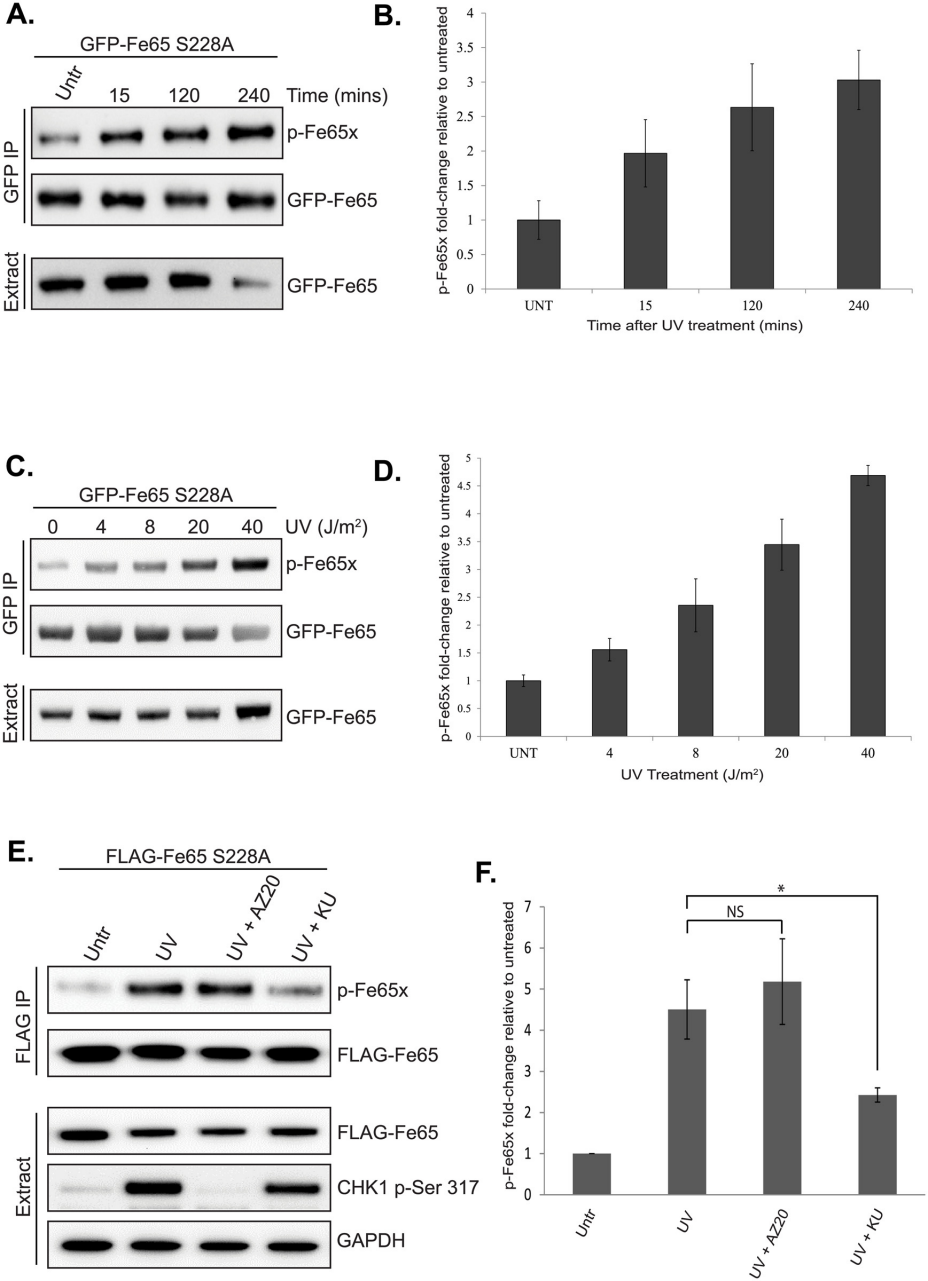
Time- and dose-dependent phosphorylation of Fe65 Ser^289^ after UV. (A) GFP-Fe65 S228A was transfected into HEK293 cells 24 hrs prior to treatment with UV (40J/m^2^). Cells were incubated for 15, 120 and 240 minutes before GFP immunoprecipitation and Western blot analysis with the indicated antibodies. (B) Quantification of p-Fe65x signal in (A) normalised to total GFP-Fe65 in corresponding immunoprecipitate. (C) GFP-Fe65 S228A was transfected into HEK293 cells 24 hrs prior to treatment with 4, 8, 20 or 40 J/m^2^ UV for 2h. After GFP immunoprecipitation, samples were analysed by Western blotting with the indicated antibodies. (D) Data in (C) was quantified as in (B). (E) HEK293 cells were transfected with FLAG-Fe65 S228A for 24h before treatment with DMSO, 5μM AZ20 or 10μM KU55933 for 45min, followed by 40J/m^2^ UV for 2h and GFP immunoprecipitation. Samples were analysed by Western blotting with the indicated antibodies and p-Fe65 quantified relative to FLAG-Fe65 in corresponding immunoprecipitates. Data is the mean and standard deviation from three independent experiments (F) Statistical significance was investigated using a two-tailed paired t-test (* p<0.05). Images are representative of at least three independent experiments.

To investigate the kinetics and dose response of Fe65 Ser^289^ phosphorylation after UV, GFP-Fe65 S228A was transfected into cells. By using GFP-Fe65 S228A for these studies, it was ensured that p-Fe65x was specifically binding only to Ser^289^ and thus data was specific for this site. Cells were exposed to the indicated doses of UV for the indicated times and GFP-Fe65 S228A immunoprecipitated before Western blotting with p-Fe65x. As shown in Fig 2A/2B, Fe65 Ser^289^ phosphorylation was increased as early as 15min after UV treatment (~2-fold) and continued to increase up to 240min (~3-fold). In Fig 2C/2D, it was demonstrated that Fe65 Ser^289^ phosphorylation was increased after UV doses as low as 4J/m^2^ (1.5-fold), and continued to increase up to 40J/m^2^ (~4.5-fold). These data confirm that Fe65 Ser^289^ is phosphorylated after UV in a dose- and time-dependent manner.

To identify the kinase that is phosphorylating Fe65 Ser^289^, specific inhibitors were used. Ser^289^ conforms to the SQ/TQ target site of the ATM/ATR protein kinases. Generally, ATR is the major kinase activated after UV, though ATM can also contribute to UV-induced DNA damage signalling. Cells were transfected with GFP-Fe65 S228A before treatment with either DMSO, AZ20 (ATR inhibitor) or KU55933 (ATM inhibitor) followed by UV and analysis of Fe65 Ser^289^ phosphorylation using p-Fe65x. As shown in Fig 2E and S2 Fig, Fe65 Ser^289^ phopshorylation was increased after UV. Interestingly, inhibition of ATR had no effect on this UV-induced phosphorylation, whilst ATM inhibition significantly reduced Ser^289^ phosphorylation (Fig 2E and 2F). To confirm the efficacy of the ATR inhibitor, the phosphorylation of a well-characterised UV-induced ATR target site (CHK1 Ser^317^) was investigated in the cell extracts. As shown in Fig 2E, CHK1 Ser^317^ phosphorylation was markedly increased after UV and completely abolished by ATR inhibition, confirming that ATR was inhibited by AZ20. Together, these data suggest that Fe65 Ser^289^ is phosphorylated after UV in an ATM-dependent manner.

### Fe65 interaction with APP is not required for Ser^289^ phosphorylation

**Fig 3:**
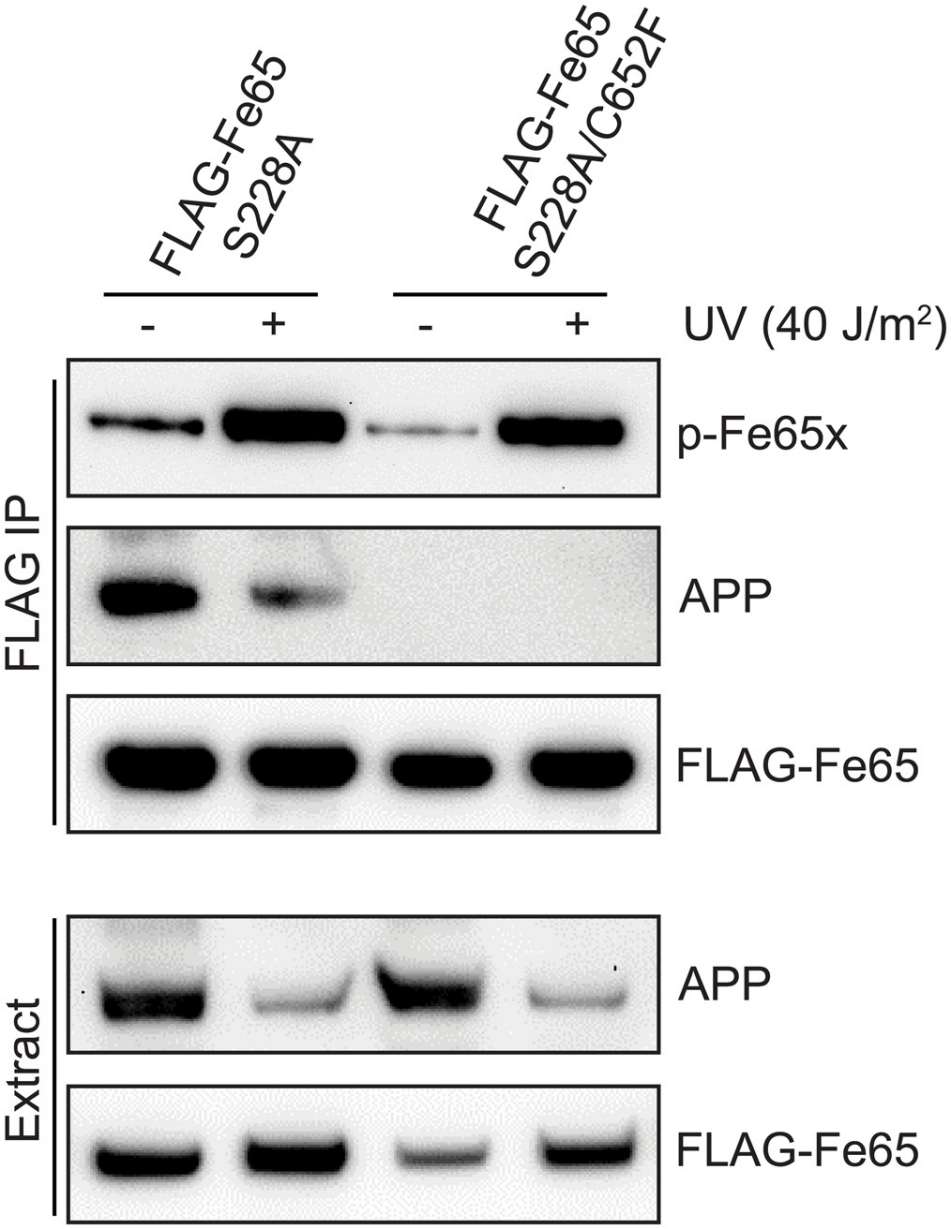
Fe65 Ser^289^ phosphorylation occurs independently of the Fe65-APP interaction. HEK293 cells were transfected with FLAG-Fe65 S228A or FLAG-Fe65 S228A/C652F for 24h before treatment with 40J/m^2^ UV for 2h. Cell extracts were subjected to FLAG immunoprecipitation before Western blot analysis with the indicated antibodies. Images are representative of at least three independent experiments.

Cells lacking Fe65 have increased sensitivity to DNA damage, which can be rescued by exogenous expression of Fe65. However, expression of a variant of Fe65 that cannot bind APP, was unable to rescue the phenotype of Fe65-deficient cells [22]. This suggests that the DNA damage response roles of Fe65 depend (at least in part) upon its interaction with APP. We therefore investigated whether the Fe65-APP interaction was required for DNA damage-induced phosphorylation of Fe65 Ser^289^. An Fe65 mutant that was unable to interact with APP was generated by mutating Cys^652^ to phenylalanine in the PTB2 domain of Fe65 (C652F). Cells were then transfected with FLAG-Fe65 S228A or FLAG-Fe65 S228A/C652F before treatment with UV, Fe65 purification and analysis of Ser^289^ phosphorylation using p-Fe65x. Again, performing all studies in a S228A mutant background ensured that results were specific to Ser^289^. As shown in Fig 3 and S3 Fig, FLAG-Fe65 S228A clearly interacted with APP in untreated cells and this interaction was abolished in the S228A/C652F mutant. Consistent with our previously published data, there was a marked decrease in the Fe65-APP complex after UV, along with a concomitant decrease in APP in the cell extracts. Experiments are in progress to determine whether the loss of the Fe65-APP complex precedes the decrease in APP protein levels or vice versa. With regards to Fe65 phosphorylation, the interaction with APP is clearly not required, with no difference in UV-induced Ser^289^ phosphorylation in FLAG-Fe65 S228A or FLAG-Fe65 S228A/C652F (Fig 3, top panel).

### Fe65 Ser^289^ phosphorylation does not regulate the Fe65-APP interaction

**Fig 4:**
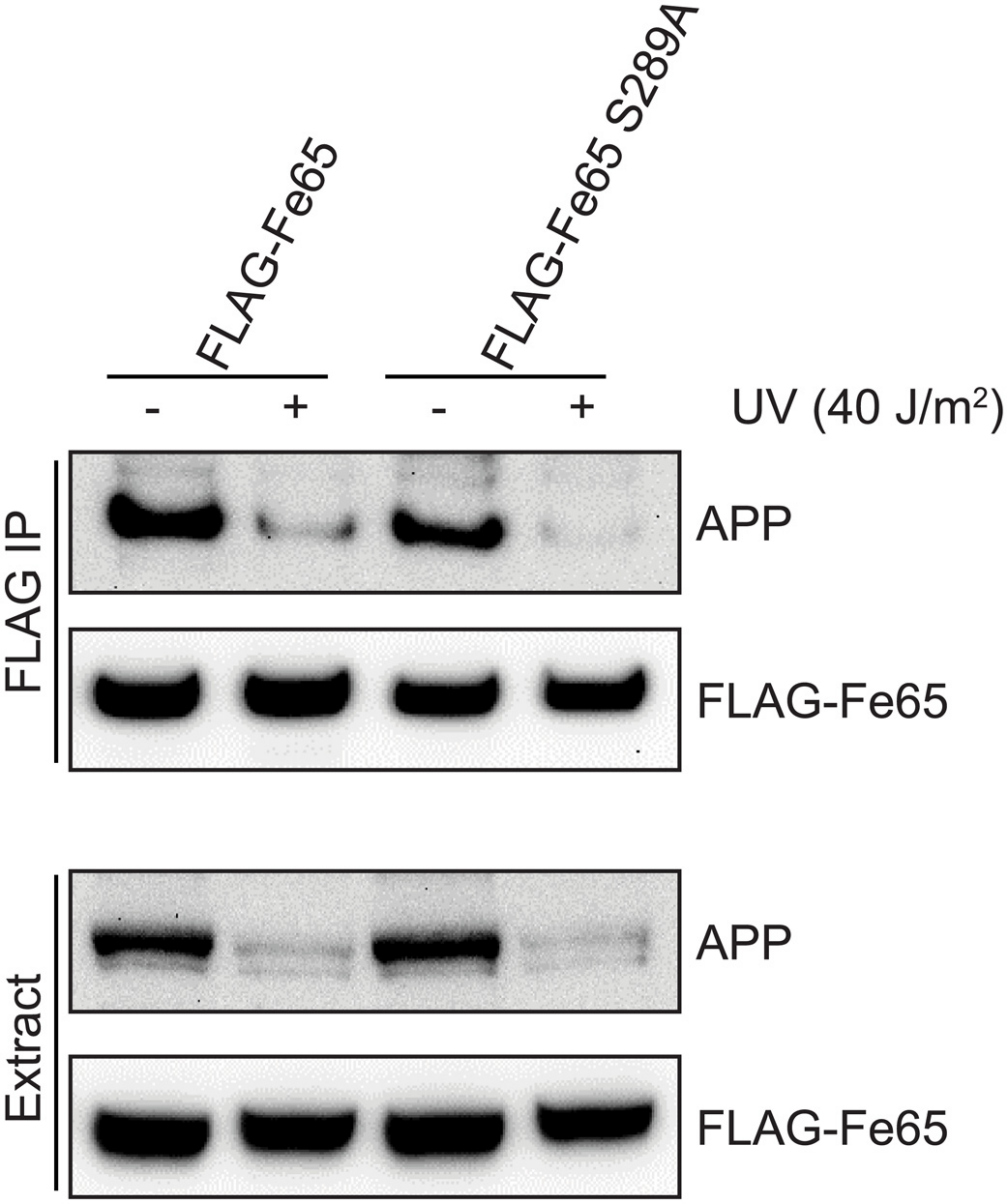
Fe65 Ser^289^ phosphorylation does not regulate the Fe65-APP interaction. HEK293 cells were transfected with FLAG-Fe65 or FLAG-Fe65 S289A for 24h before treatment with 40J/m^2^ UV for 2h. Cell extracts were subjected to FLAG immunoprecipitation before western blot analysis with the indicated antibodies. Images are representative of at least three independent experiments.

The Fe65-APP interaction can be disrupted by APP Thr^668^ phosphorylation. To investigate whether Fe65 Ser^289^ phosphorylation affected the interaction with APP, we compared the interaction between FLAG-Fe65 or FLAG-Fe65 S289A and endogenous APP, before and after UV-induced DNA damage. As shown in Fig 4 and S4 Fig, Fe65 interacted with APP in untreated cells, with levels of the Fe65-APP complex being markedly reduced by UV treatment, in agreement with our previously published data. Again, there was a concomitant decrease in the protein levels of APP in cell extracts after UV. Mutation of Ser^289^ did not affect the interaction with APP in untreated cells, or after UV treatment. These data show that Ser^289^ phosphorylation does not regulate the Fe65-APP interaction and is not involved in the UV-induced decrease in the Fe65-APP complex (or the concomitant decrease in APP levels in the cell extracts after UV).

### Mutation of Fe65 Ser^289^ has no effect on Fe65-APP-mediated gene transcription

**Fig 5:**
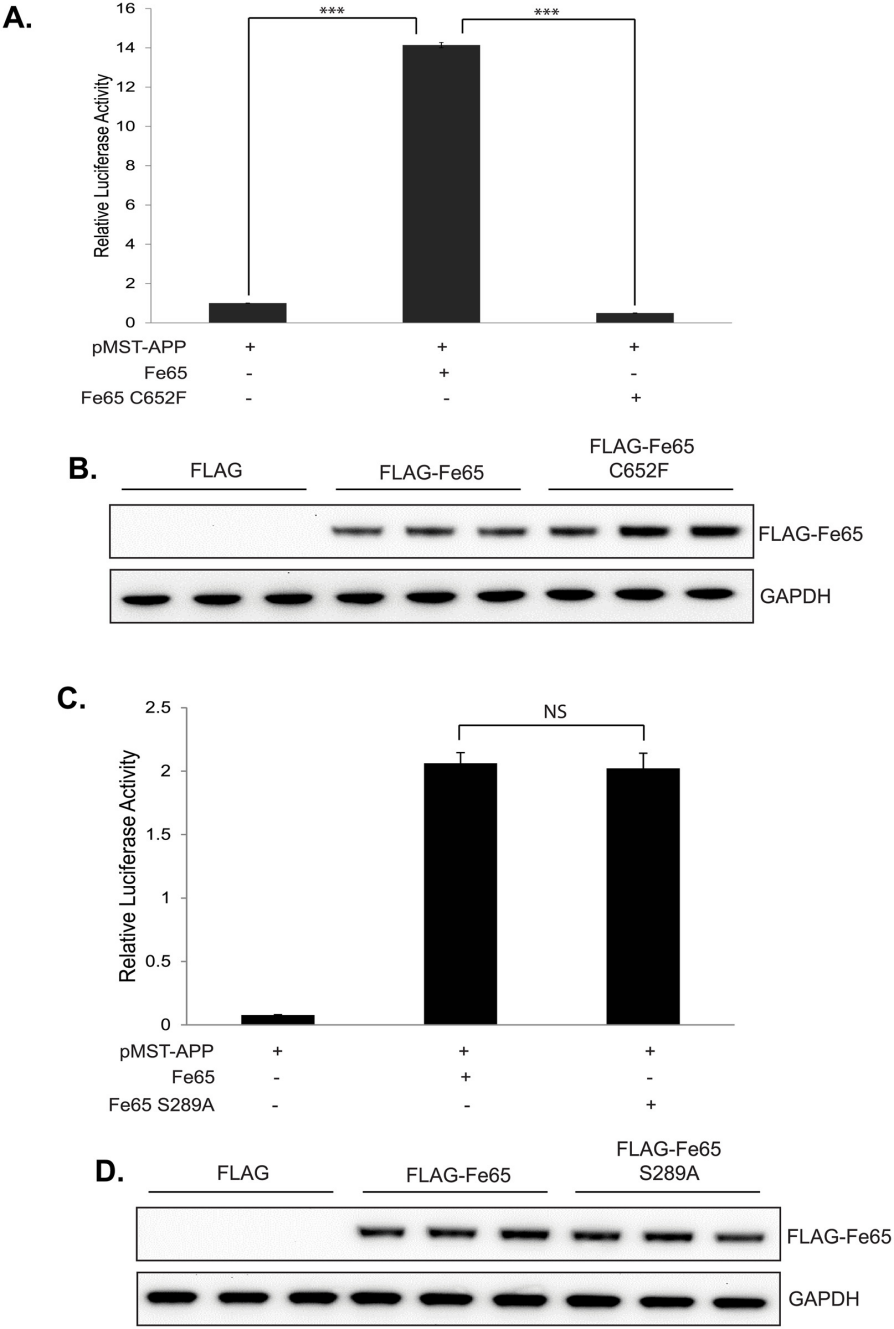
Fe65 Ser^289^ does not regulate Fe65-APP transcriptional activity. (A) HEK293 cells were transfected with the indicated plasmids for 24h prior to cell lysis and measurement of both firefly and renilla luciferase activities. To normalise for transfection efficiency the ratio of firefly/renilla luciferase values were calculated (each sample in triplicate). (B) The cellular samples used for the luciferase assay in (A) were analysed by Western blotting to confirm equal expression of both FLAG-Fe65 and FLAG-Fe65 C652F. (C) and (D) HEK293 cells were transfected with the indicated plasmids for 24h prior to luciferase assays and Western blot analysis as in (A) and (B). Data is the mean and standard deviation of triplicate samples and is representative of at least three independent experiments. The significance of the observed changes in relative luciferase activity were investigated using a paired t-test (two-tailed). Levels of significance were defined as follows: p < 0.05 (*), p < 0.01 (**) and p < 0.001 (***).

Previous studies have demonstrated that Fe65 markedly enhanced APP-mediated gene transcription. To investigate the potential role of Fe65 Ser^289^ phosphorylation in Fe65-APP transcriptional activity, a luciferase-based reporter gene assay was used in HEK293 cells. This system has been used in multiple studies and has revealed a complex regulatory network controlling Fe65-APP mediated transcription [12,17,19,30]. To validate the system in the present study, we showed that pMST-APP (full-length APP fused to the GAL4 DNA binding domain) resulted in little luciferase activity (firefly luciferase under the control of a GAL4 binding site, Fig 5A). Importantly, expression of FLAG-Fe65 markedly increased APP transcriptional activity (~14-fold, whereas FLAG-Fe65 C652F, unable to bind APP, did not (Fig 5A). Western blotting confirmed expression of both FLAG-Fe65 and FLAG-Fe65 C652F (Fig 5B). A previous study has demonstrated that Fe65 expressed without pMST-APP does not result in luciferase transcription in this system [30]. Having validated the luciferase assay, the effect of mutating Ser^289^ in Fe65 was investigated. As shown in Fig 5C/5D, FLAG-Fe65 and FLAG-Fe65 S289A were both able to activate APP transcriptional activity to the same extent. These studies suggest that Fe65 Ser^289^ phosphorylation is not involved in regulating the transcriptional activity of the Fe65-APP complex.

To confirm the reproducibility of data, repeats of relevant studies are included in the supplementary material (S1–S4 Figs).

## Discussion

In the present study, we identified Fe65 Ser^289^ as a novel site of UV-induced phosphorylation. Phosphorylation increased in a dose- and time-dependent manner and was found to be targeted by ATM, rather than ATR, after UV in an APP-independent manner. Mutation of Ser^289^ to Ala did not affect the Fe65-APP interaction nor the Fe65-APP transcriptional activity. These findings are in contrast to the Fe65 Ser^228^ site that we previously identified, which we found to be targeted by ATR after UV and contributed to the regulation of Fe65-APP transcriptional activity.

Previous studies demonstrated that Fe65 displayed a phosphatase-sensitive gel mobility shift after DNA damage [20]. Our recent research has aimed to identify these specific sites, with Ser^228^ described recently and Ser^289^ identified in the present study [17]. These sites both conform to the SQ/TQ consensus sequence for the ATM/ATR kinases and are relatively close together, in terms of the amino acid sequence of Fe65. In total, Fe65 contains 10 SQ/TQ sites, and 4 of these are found between residues 228 and 300, a region including the functionally important WW domain, which mediates Fe65 interactions with several binding partners. Often, ATM and ATR have been found to target ‘clusters’ of SQ/TQ sites, with multiple phosphorylation sites identified in close sequence proximity [26]. Given our recent data, it is tempting to speculate that ATM/ATR target a cluster of SQ/TQ sites within Fe65 228–300, and this in turn could regulate WW domain-dependent interactions with several proteins including Mena, c-abl and the nucleosome assembly factor SET [10,31,32]. Immunoprecipitation studies with relevant phosphomutants are in progress to test this hypothesis. Interestingly, whilst both of these sites were phosphorylated after UV, they appear to be differentially regulated, with Ser^228^ being ATR-dependent and Ser^289^ being ATM-dependent. To gain further insight into this differential regulation, it will be important to compare the phosphorylation kinetics of each site after different forms of DNA damage and investigate the kinase involved.

Fe65 knockout (KO) mouse embryonic fibroblasts (MEFs) showed elevated levels of DNA damage after exposure to etoposide or ionising radiation, caused (at least in part) by inefficient recruitment of the histone acetyltransferase TIP60 to sites of DNA double strand breaks (DSBs), inefficient histone H4 acetylation and defective chromatin relaxation (an essential part of DNA repair processes) [20,22]. Whilst exogenous expression of Fe65 could correct the phenotype of Fe65 KO MEFs, a mutant version that was unable to interact with APP could not. This suggests that the interaction of Fe65 with APP is involved in the DNA damage response roles of Fe65, possibly involving a conformational change of Fe65. Cao and Suddhof suggested that free Fe65 adopts a ‘closed’ conformation, due to intramolecular interactions between the WW and PTB domains [12]. Upon binding to APP, these interactions are disrupted and Fe65 converts to an ‘open’ conformation, which is able to bind chromatin and initiate remodelling. In support of this, it was demonstrated that wild-type Fe65 could bind chromatin, whereas a non APP-interacting mutant version of Fe65 could not [22]. Our data has shown that UV-induced phosphorylation of Fe65 on Ser^228^ and Ser^289^ occurs independently of the APP interaction. Assuming that the above model is correct, this suggests that the ‘closed’ conformation of Fe65 can still be targeted by the ATM/ATR kinases and that the phosphorylation occurs independently of chromatin interaction. Interestingly, as well as multiple studies demonstrating that Fe65 contributes to the cellular DNA damage response, recent publications have implicated Fe65 in breast cancer biology. More specifically, Sun et al (2014) provided some evidence that Fe65 expression is increased in breast cancer cell lines and that Fe65 was able to act as an activator of estrogen receptor-α (ERα), promoting cell growth [33]. Interestingly, Fe65 overexpression also decreased the antagonistic activity of tamoxifen, identifying Fe65 as a potential novel factor in mediating tamoxifen resistance in breast cancer. The same research group have recently demonstrated that Fe65 can suppress cell migration and invasion in ERα-negative breast cancer cells by recruiting Tip60 to cortactin and stimulating its acetylation [34]. Given the role of Fe65 in the DNA damage response and the recent studies mechanistically linking Fe65 to breast cancer, it will be important to elucidate the functional significance of DNA damage-induced phosphorylation of Fe65 (on both Ser^228^ and Ser^289^) in these particular research areas. Studies are in progress to functionally characterise these new phosphorylation sites in response to DNA damage by expressing phosphomutant versions of each in Fe65-deficient cells.

Mutation of Ser^289^ to Ala did not affect the Fe65-APP interaction or the transcriptional activity of this complex. On the contrary, we previously demonstrated using both phosphoblocking and phopshomimetic mutations, that Fe65 Ser^228^ phosphorylation inhibited Fe65-APP transcriptional activity [17]. In addition, we have repeatedly shown that DNA damage caused a loss of the Fe65-APP complex, with a concomitant decrease in the protein levels of APP. It is unclear whether UV-induced loss of the Fe65-APP complex precedes APP protein depletion or vice versa. Previous studies showed down-regulation of APP in response to both UV and camptothecin, with Cuesta et al (2009) suggesting a p53-dependent reduction in the transcription of APP, whereas Almenar-Queralt (2014) showed that the decrease in APP was due to secretase-mediated cleavage [7,23,35]. Our unpublished studies demonstrate that the decrease in both the Fe65-APP complex and APP in cell extracts occurs within 30 minutes after UV, suggesting that the effect is not caused by altered transcription. The mechanisms mediating APP depletion after DNA damage, and whether Fe65 contributes to this effect, require further study. It will also be important to understand the precise fate of APP after different forms of DNA damage. In particular, if APP loss is secretase-dependent, this could have important consequences in terms of Aβ production and thus be directly relevant to AD aetiology. In addition, loss of the Fe65-APP complex after DNA damage is likely to have a significant impact on the transcription of APP target genes. It has been proposed that the cytotoxicity of Aβ is mediated (at least in part) by inducing mitochondrial dysfunction and increased oxidative stress. Among multiple cellular targets, reactive oxygen species can cause DNA damage and we are therefore currently investigating whether oxidative stress and/or Aβ peptides affect Fe65 phosphorylation and cellular levels of the Fe65-APP complex.

Given the roles of Fe65 in Alzheimer’s biology and DNA damage response pathways, it is important to fully elucidate the mechanisms regulating Fe65 and how these impact on the function of Fe65 and Fe65-binding partners within the cell. This study has identified Ser^289^ as a novel site of ATM-dependent phosphorylation after UV-induced DNA damage. Whilst this site appears to have no role in mediating the Fe65-APP interaction or transcriptional activity of this complex, it will be important to investigate how this site contributes to the DNA damage response roles of Fe65.

## Supporting Information

S1 Fig P-Fe65x detects Ser^228^ and at least one other phosphorylation site. (A) HEK293 cells were transfected with GFP-Fe65 or GFP-Fe65 S228A for 24h before treatment with 40J/m^2^ UV for 2h. Cell extracts were subjected to GFP immunoprecipitation and samples analysed by Western blotting using the indicated antibodies. Please note, we have previously published the data in S1A (see Jowsey and Blain, 2015). (B) HEK293 cells were transfected with GFP-Fe65 S228A 1–349 for 24h before treatment with 40J/m^2^ UV for 2h. After GFP immunoprecipitation, each sample was split and half treated with lambda phosphatase for 15min at 30C, before western blot analysis with the indicated antibodies.
(PDF)

S2 Fig ATM-medicated phosphorylation of Fe65 Ser^289^ after UV. HEK293 cells were transfected with FLAG-Fe65 S228A for 24h before treatment with DMSO, 5μM AZ20 or 10μM KU55933 for 45min, followed by 40J/m^2^ UV for 2h and GFP immunoprecipitation. Samples were analysed by Western blotting with the indicated antibodies.
(PDF)

S3 Fig Fe65 Ser^289^ phosphorylation occurs independently of the Fe65-APP interaction. HEK293 cells were transfected with FLAG-Fe65 S228A or FLAG-Fe65 S228A/C652F for 24h before treatment with 40J/m^2^ UV for 2h. Cell extracts were subjected to FLAG immunoprecipitation before Western blot analysis with the indicated antibodies.
(PDF)

S4 Fig Fe65 Ser^289^ phosphorylation does not regulate the Fe65-APP interaction. HEK293 cells were transfected with FLAG-Fe65 or FLAG-Fe65 S289A for 24h before treatment with 40J/m^2^ UV for 2h. Cell extracts were subjected to FLAG immunoprecipitation before western blot analysis with the indicated antibodies.
(PDF)
