# Supplementary figures and images for: Fe65 Is Phosphorylated on Ser289 after UV-Induced DNA Damage
Source: PLoS One. 2016 May 13;11(5):e0155056. doi: 10.1371/journal.pone.0155056 (PMC4866770; doi:10.1371/journal.pone.0155056)

# S1 Fig

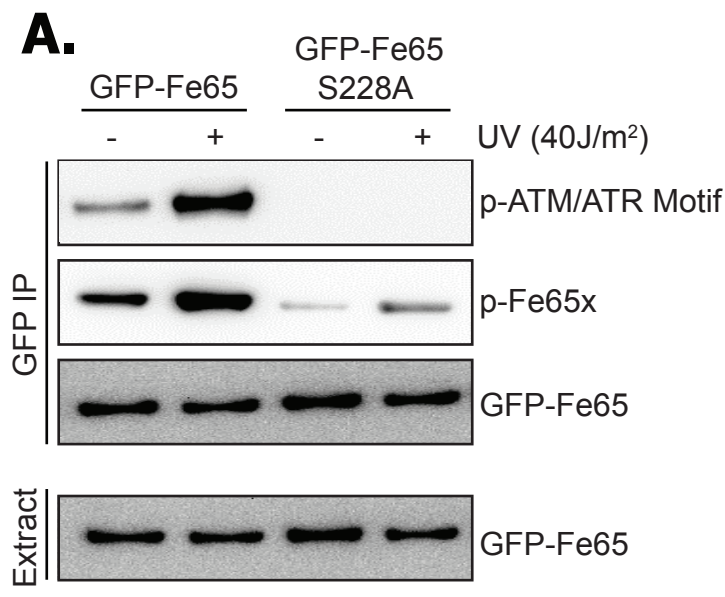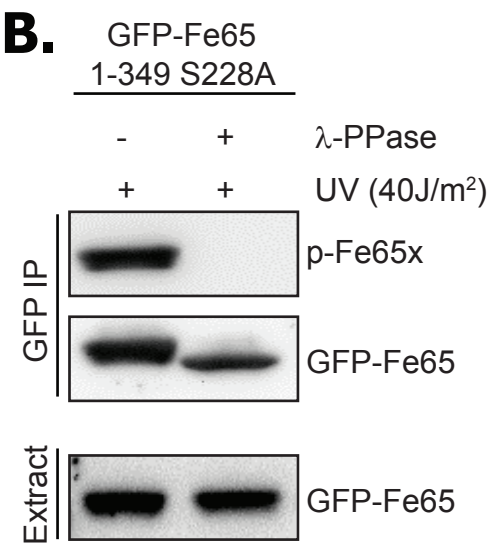

Supplement: S1 Fig — (A) HEK293 cells were transfected with GFP-Fe65 or GFP-Fe65 S228A for 24h before treatment with 40J/m2 UV for 2h. Cell extracts were subjected to GFP immunoprecipitation and samples analysed by Western blotting using the indicated antibodies. Please note, we have previously published the data in S1A (see Jowsey and Blain, 2015). (B) HEK293 cells were transfected with GFP-Fe65 S228A 1–349 for 24h before treatment with 40J/m2 UV for 2h. After GFP immunoprecipitation, each sample was split and half treated with lambda phosphatase for 15min at 30C, before western blot analysis with the indicated antibodies. (PDF) [file pone.0155056.s001.pdf]

S2 Fig

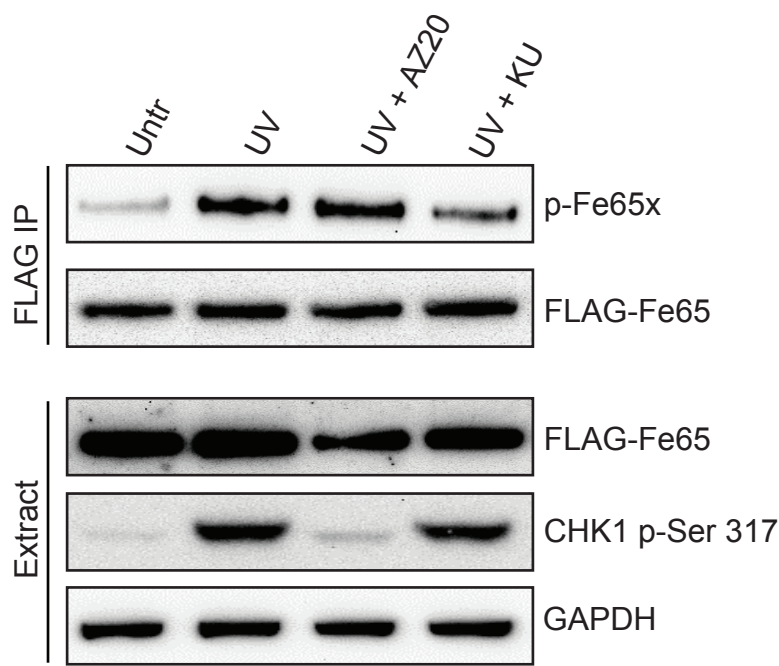

Supplement: S2 Fig — HEK293 cells were transfected with FLAG-Fe65 S228A for 24h before treatment with DMSO, 5μM AZ20 or 10μM KU55933 for 45min, followed by 40J/m2 UV for 2h and GFP immunoprecipitation. Samples were analysed by Western blotting with the indicated antibodies. (PDF) [file pone.0155056.s002.pdf]

S3 Fig

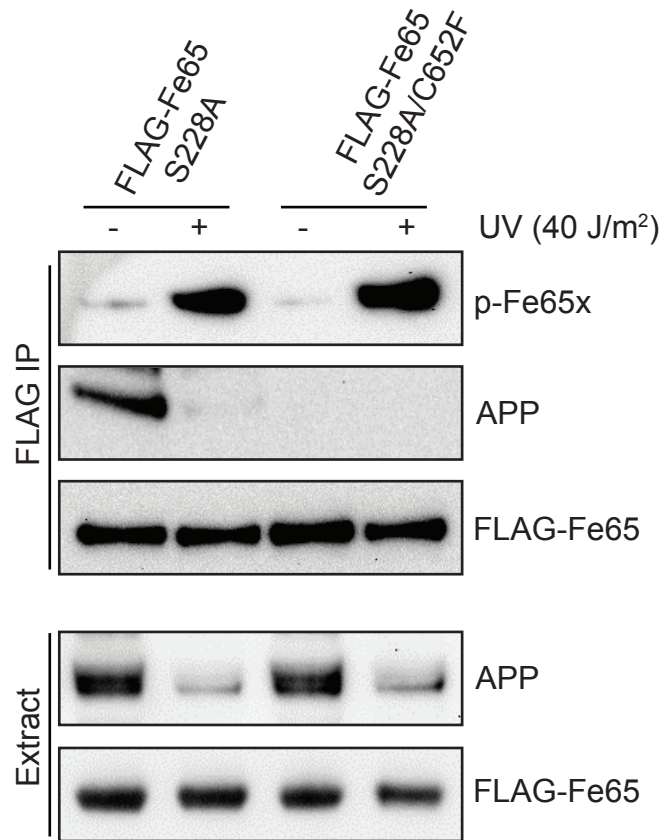

Supplement: S3 Fig — HEK293 cells were transfected with FLAG-Fe65 S228A or FLAG-Fe65 S228A/C652F for 24h before treatment with 40J/m2 UV for 2h. Cell extracts were subjected to FLAG immunoprecipitation before Western blot analysis with the indicated antibodies. (PDF) [file pone.0155056.s003.pdf]

S4 Fig

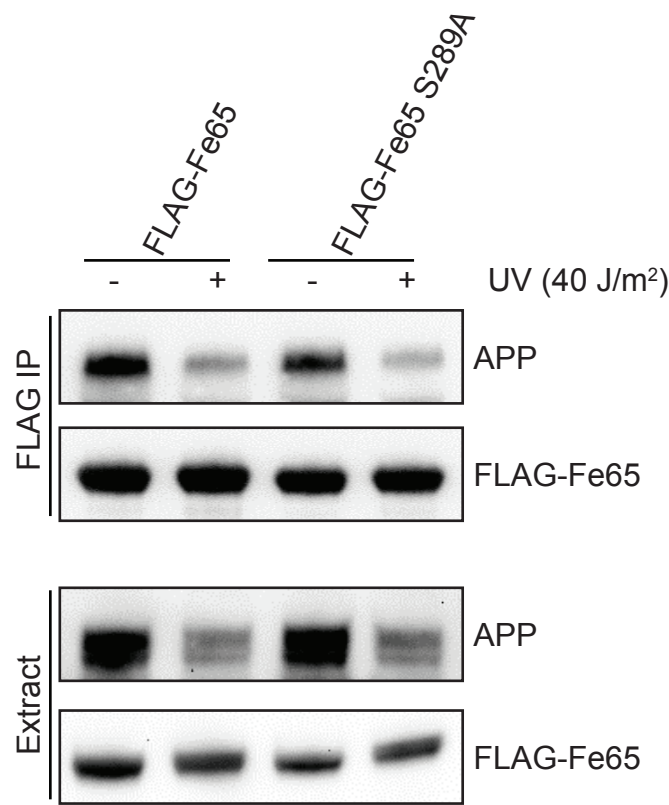

Supplement: S4 Fig — HEK293 cells were transfected with FLAG-Fe65 or FLAG-Fe65 S289A for 24h before treatment with 40J/m2 UV for 2h. Cell extracts were subjected to FLAG immunoprecipitation before western blot analysis with the indicated antibodies. (PDF) [file pone.0155056.s004.pdf]
